# Benefits of normal body mass index on physical fitness: A cross-sectional study among children and adolescents in Xinjiang Uyghur Autonomous Region, China

**DOI:** 10.1371/journal.pone.0220863

**Published:** 2019-08-15

**Authors:** Cunjian Bi, Junmin Yang, Jian Sun, Yi Song, Xiaoyan Wu, Feng Zhang

**Affiliations:** 1 School of Physical Education and Health, East China Normal University, Shanghai, China; 2 School of Physical Education, Xihua University, Chengdu, China; 3 Institute of Physical Education, Xinjiang Normal University, Urumqi, China; 4 Institute of Child and Adolescent Health, Peking University, Beijing, China; School of Public Health Peking University, Beijing, China; 5 Department of Maternal, Child & Adolescent Health, School of Public Health, Anhui Medical University,Hefei, China; University of Brasilia, BRAZIL

## Abstract

**Objective:**

To assess the association between body mass index (BMI) and physical fitness index (PFI) among children and adolescents in Xinjiang Uyghur Autonomous Region, China.

**Methods:**

In total, 17,618 children and adolescents aged 7–18 years from the selected region were enrolled in this cross-sectional study (8,800 boys and 8,818 girls). Participants were stratified by age and sex and divided into five groups by BMI percentiles: very low (BMI <5th percentile); low, (5th ≤ BMI < 15th percentile); normal (15th ≤ BMI < 85th percentile); high (85th ≤ BMI < 95th percentile); and very high (BMI ≥95th percentile). PFI was assessed by height, weight, and five health-related fitness indicators (grip strength, standing long jump, sit and reach, 50 m dash, and endurance run).

**Results:**

BMI was significantly associated with PFI during adolescence (13–18 years) in boys and pre-adolescence (7–12 years) in girls. Between the ages of 13 and 18 years, an increase in BMI had a greater impact on PFI in boys than girls. PFI showed a parabolic curvilinear relationship with BMI.

**Conclusion:**

Children and adolescents in China’s Xinjiang Uyghur Autonomous Region with a normal BMI demonstrated good physical fitness. A BMI below or above the normal range may contribute to poor physical fitness. The relationship between BMI and PFI shows an inverted U-shaped curve.

## Introduction

Physical fitness commonly includes cardiorespiratory endurance, muscle strength endurance, flexibility, and body composition[1]. Currently, physical fitness is attracting extensive research attention worldwide, and has been shown to be positively associated with cognition[2], weight status[3], psychological well-being[4], academic achievement scores[2, 5], and performance on real-world tasks[6]. In contrast, low physical fitness may increase the prevalence of risk factors for cardiovascular disease in childhood, potentially tracking into adulthood[7]. Many investigations into factors influencing the physical fitness of youth used body mass index (BMI) as a surrogate for body composition[3]. Although the rising trends in BMI among children and adolescents in many high-income countries have plateaued, they have accelerated in parts of Asia, including China[8]. This has resulted in a significant public health problem. In particular, being overweight in childhood and adolescence is associated with earlier onset of chronic disorders such as type 2 diabetes[9].

Research suggests the relationship between BMI and physical fitness has been considered from three main perspectives. First, most studies that focused on overweight/obese individuals showed a negative linear relationship between BMI and physical fitness[10–12]. Second, some researchers argue that BMI is a potential covariate of physical fitness[13, 14]. Third, other studies suggest that the relationship between the physical fitness index (PFI) and BMI is quadratic[15–18] during adolescence. However, irrespective of these different perspectives, few prior studies have focused on multi-ethnic populations.

China is a multi-ethnic Asian country with 55 ethnic minorities. Because of these ethnic and cultural subgroups, the mean/median BMI in China is lower than that in non-Asian populations[19]. This may mean, even though at similar BMI levels, Asian populations have a higher percentage of body fat and less lean mass than other races [20]. Xinjiang Uyghur Autonomous Region is the largest province in China. It is located in the far northwest and covers approximately one-sixth of China’s total landmass. However, the region is economically underdeveloped because of poor communications. There are more than 13 ethnic minorities living in Xinjiang Uyghur Autonomous Region. The largest minority group is the Uyghur, as implied by the region’s name[21]. The Uyghur have a mixture of European (60%) and East Asian ancestry (40%), and differ from Han peoples in lifestyle, religion, culture, diet, and genetic background[22]. For example, the primary foods for the Uyghur are wheat, beef, mutton, and dairy products, which are high-fat foods.

As a multi-ethnic region, Xinjiang offers a good opportunity to examine the diversity in the relationship between BMI and physical fitness. Because of its location in far northwestern China and limited public health resources, few studies have assessed the relationship between BMI and physical fitness in the region, especially among children and adolescents. In total, 17,618 children and adolescents (aged 7–18 years) from this region were evaluated in the present study. This allowed us to analyze the cross-sectional relationship between BMI and PFI.

## Methods

The data were from a provincial survey in Xinjiang, which was extracted from the 2014 Chinese National Survey on Students’ Constitution and Health (CNSSCH). The It has been conducted every five years since 1985 by China’s Ministry of Education, State Sports General Administration, National Health and Family Planning Commission, National Civil Affairs Commission, Ministry of Science and Technology, and Ministry of Finance[23]. It is, so far, the largest nationally representative sample of school-age children and adolescents in China. The present study was approved by the Medical Research Ethics Committee of Peking University Health Science Center(IRB00001052-18002).

Three socioeconomic classes (“upper”, “moderate” and“low”) was defined base on the regional gross domestic product, total yearly income per capita, average food consumption per capita, the natural growth rate of population, and the regional social welfare index. In each class, there are four populations with an equal size of sample including urban and rural males and females, which was stratified by sex and area of residence. Taking into account the socio-economic situation and the national distribution, Children and adolescents from six cities of Xinjiang, including Urumqi, Yining, Akesu, Kashgar, Kizilesu Kirgiz Autonomous Prefecture and Altay Prefecture, were selected using a multistage stratified cluster sampling design. We excluded 204 participants (1.14%) because of missing data or extreme values. The extreme boundary values for each indicator were: BMI ≤10 or >40 kg/m^2^; standing long jump <50 or >300 cm; sit and reach ≤−8 or >26 cm; 50 m dash <6.0 or >16.0 s; and endurance run: 50 m × 8 round-trips <60 or >200 s, 800 m run <140 or >400 s, and 1,000 m run <150 or >370 s[23]. Finally, 17,618 multi-ethnic children and adolescents from Xinjiang aged 7–18 years (8,800 boys and 8,818 girls) were included in this study (Table 1).

**Table 1:** Gender distribution by age in participating children and adolescents from Xinjiang, China.

Age(yr)	Boys	Girls	Total
7	754	754	1508
8	723	734	1457
9	748	737	1485
10	736	757	1493
11	735	656	1391
12	715	725	1440
13	693	738	1431
14	736	729	1465
15	741	733	1474
16	750	754	1504
17	733	758	1491
18	736	743	1479
Total	8800	8818	17618

In order to ensure the safety and accuracy of the measurements during the investigation. All the tests used the same type of instruments according to the standard procedures required by 2014 CNSSCH. The measurement instruments were calibrated before use and administered by investigators who were trained before the investigation and qualified to administer the instruments. Each test was completed at a fixed time of the day to reduce data deviation caused by different test times.

Participants’ height and weight were measured according to the 2014 CNSSCH requirements and instruments[23]. Subjects were required to wear only light clothes and stand straight, barefoot and at ease when being measured. Weight was measured to the nearest 0.1 kg with a standardized scale and height to the nearest 0.1 cm with a portable stadiometer. BMI was calculated as body weight (kg)/height (m^2^). Five physical fitness indicators were considered in the present study: grip strength, standing long jump, sit and reach, 50 m dash, and endurance run. All physical examinations were performed separately for boys and girls as follows.

1. Grip strength was measured to the nearest 0.1 kg by a handgrip dynamometer. Participants stood upright with their feet shoulder-width apart, arms dropped obliquely, and palms inward. They then squeezed the dynamometer twice, as hard as they could. The larger value was recorded.
2. Standing long jump was measured with a distance of at least 30 cm between the starting line and pit. Each participant stood behind the starting line with their feet apart at a natural distance. They were instructed to jump horizontally forward as far as possible, taking off with both feet. The distance from the starting line to the heel of the foot closest to the start line was recorded. Each participant completed two trials, and the greater distance was recorded.
3. Sit and reach was scored as the most distant point (nearest centimeter) reached with the fingertips. The participant sat on a mat with shoes removed, knees straight, and toes separated naturally, with the heels together and placed on the pad of the instrument. The height of the guide rail was adjusted to keep the participant’s toes even with the lower edge of the marker. The participant was then instructed to slowly reach forward and push the marker forward with the middle fingertips of both hands as far as possible on the scale. Two trials were completed, and the greater distance was recorded.
4. The 50 m dash involved participants running toward the finish line as fast as they could from a standing start immediately on hearing the starting signal. The time taken to run 50 m was measured in seconds (to the nearest 0.1s).
5. The endurance run comprised eight 50 m round-trips for those aged 6–12 years, an 800 m run for girls aged 13–18 years, and a 1,000 m run for boys aged 13–18 years. In the eight 50 m round-trips, there were posts at the start and finish lines. On hearing the starting signal, participants ran (from a standing start) toward the finish line, bypassed the post counterclockwise and ran back to the start line. This process represented one completed lap, and was repeated three more times. The time taken was recorded in seconds (to the nearest 0.1s). The 800 and 1,000 m runs were performed according to the rules for the 50 m dash.

After stratification by age and sex, participants were divided into five BMI groups by percentile: very low, BMI < 5th percentile; low, 5th ≤ BMI < 15th percentile; normal, 15th ≤ BMI < 85th percentile; high, 85th ≤ BMI < 95th percentile; and very high, BMI ≥ 95th percentile. The BMI-Z score was calculated for each group. In addition, children and adolescents were categorized into four age groups: lower primary school age (7–9 years); upper primary school age (10–12 years); middle school age (13–15 years); and high school age (16–18 years).

Five test indicators were standardized based on age and sex. The Z-score was calculated as Z-score = (measured value of each physical fitness index − mean value of each physical fitness index) / standard deviation of physical fitness indicator in each group. The Z-scores for these indicators were summed to obtain the PFI[24]. The Z-scores for the 50 m dash and endurance run were reversed, because lower times reflect better performances in these two tests. Therefore, PFI = Z grip strength + Z standing long jump + Z sit and reach–Z 50 m dash − Z endurance run.

Pearson’s correlations among the five health-related physical fitness indicators in different age groups ranged from 0.015–0.530. There were no significant correlations between sit and reach and 50 m dash among boys aged 7–9 years, grip strength and endurance run in girls aged 10–12 years, sit and reach and endurance run in girls aged 10–12 years, or sit and reach and endurance run in girls aged 16–18 years. Other indicators showed statistically significant correlations (*P* < 0.01) (Table 2). This suggested there were close relationships among various physical fitness indicators, and the PFI reflected the overall physical fitness of participating children and adolescents.

**Table 2:** Correlations between health test indicators for participating children and adolescents by age group (*r*-value).

Age(yr)	A/B	A/C	A/D	A/E	B/C	B/D	B/E	C/D	C/E	D/E
Boys										
7yr-9yr	0.275^a^	0.028	-0.219^a^	-0.071^a^	0.182^a^	-0.441^a^	-0.355^a^	-0.026	-0.069^a^	0.388^a^
10yr-12yr	0.303^a^	0.091^a^	-0.251^a^	-0.103^a^	0.213^a^	-0.530^a^	-0.419^a^	-0.088^a^	-0.114^a^	0.437^a^
13yr-15yr	0.418^a^	0.172^a^	-0.333^a^	-0.155^a^	0.181^a^	-0.401^a^	-0.310^a^	-0.168^a^	-0.143^a^	0.388^a^
16yr-18yr	0.259^a^	0.090^a^	-0.156^a^	-0.070^a^	0.082^a^	-0.307^a^	-0.252^a^	-0.113^a^	-0.151^a^	0.262^a^
Girls										
7yr-9yr	0.276^a^	0.110^a^	-0.246^a^	-0.106^a^	0.214^a^	-0.423^a^	-0.382^a^	-0.129^a^	-0.105^a^	0.341^a^
10yr-12yr	0.304^a^	0.173^a^	-0.281^a^	-0.029	0.226^a^	-0.479^a^	-0.219^a^	-0.167^a^	-0.038	0.217^a^
13yr-15yr	0.238^a^	0.129^a^	-0.205^a^	-0.152^a^	0.145^a^	-0.385^a^	-0.280^a^	-0.124^a^	-0.118^a^	0.433^a^
16yr-18yr	0.189^a^	0.102^a^	-0.174^a^	-0.125^a^	0.063^a^	-0.412^a^	-0.173^a^	-0.077^a^	-0.015	0.289^a^

Note

^a^ <0.01; A, grip strength; B, standing long jump; C, sit and reach; D, 50 m dash; E, endurance run.

Comparisons of PFI between different BMI groups for boys and girls were performed using one-way analysis of variance. The effect size for the difference between the low and high groups was calculated by Cohen’s d (small effect = 0.2; medium effect = 0.5; large effect = 0.8)[25]. The relationships between BMI and PFI for different age groups (7–9, 10–12, 13–15, and 16–18 years) were investigated using a nonlinear regression model (PFI = aBMI^2^ + bBMI + c; where a, b, c were constants). PFI was used as the dependent variable, and BMI was considered the independent variable. The level of statistical significance was set at 0.05. All analyses were conducted using SPSS version 23.0 (SPSS Inc., Chicago, IL, USA).

## Results

**Fig 1:**
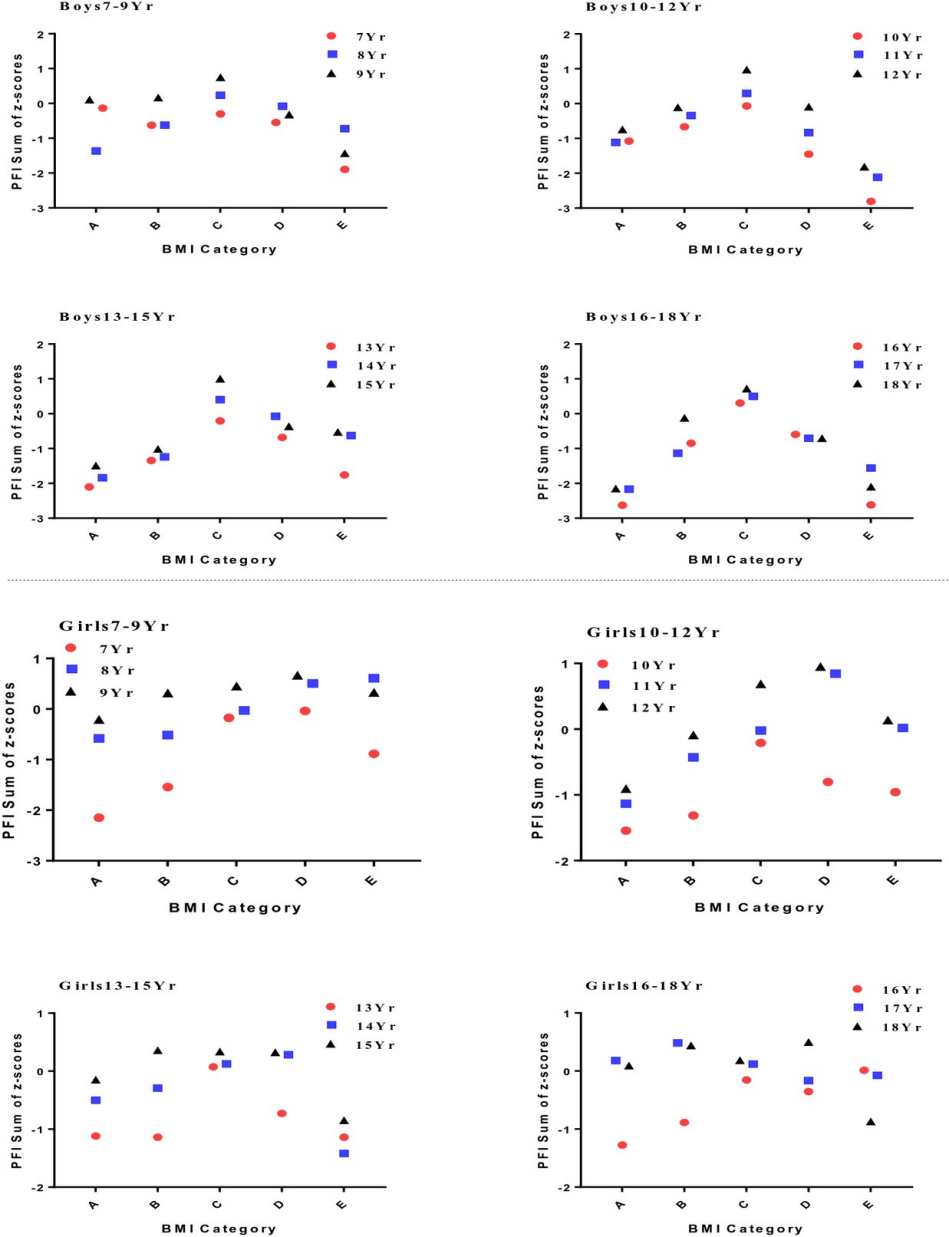
Trends in physical fitness index and body mass index for participating children and adolescents by age group. **A:**BMI<5th Percentile; **B:** 5th≤BMI<15th Percentile; **C:** 15th≤BMI<85th Percentile; **D:** 85th≤BMI<95th Percentile; and **E:** BMI≥95th Percentile.

The averages and standard deviations for PFI by BMI categories for boys and girls are shown in Tables 3 and 4. Overall, the average PFI for boys in the normal BMI group was higher than that for boys in the very low, low, high, and very high BMI groups, with significant differences observed for some age groups. A parabolic trend of increasing and then decreasing BMI-Z score was observed in most age groups (Fig 1).

**Table 3:** Comparisons of physical fitness index among boys in different body mass index and age groups.

Age(yr)	BMI<5th Percentile(A)		5th≤BMI<15th Percentile(B)		15th≤BMI<85th Percentile(C)		85th≤BMI<95th Percentile(D)		BMI≥95th Percentile(E)		Cohen’s *d* ^*#*^									
	N	Mean(SD)	N	Mean(SD)	N	Mean(SD)	N	Mean(SD)	N	Mean(SD)	A/B	A/C	A/D	A/E	B/C	B/D	B/E	C/D	C/E	D/E
7	37	-0.134(2.195)	76	-0.629(2.725)	528	-0.301(2.817)	76	-0.546(3.212)	37	-1.894(3.444)	0.2	0.1	0.2	0.6^a^	0.1	0.0	0.4^a^	0.1	0.5^a^	0.4^a^
8	36	-1.363(3.101)	72	-0.625(3.002)	507	0.235(2.963)	72	-0.081(3.112)	36	-0.729(2.840)	0.2	0.5^a^	0.4^a^	0.2	0.3^a^	0.2	0.0	0.1	0.3	0.2
9	37	0.108(3.022)	75	0.164(3.043)	524	0.746(3.032)	75	-0.322(3.234)	37	-1.434(3.273)	0.0	0.2	0.1	0.5^a^	0.2	0.2	0.5^a^	0.3^a^	0.7^a^	0.3
10	36	-1.078(2.524)	74	-0.664(3.045)	516	-0.067(2.887)	74	-1.451(2.877)	36	-2.807(2.852)	0.2	0.4^a^	0.1	0.6^a^	0.2	0.3	0.7^a^	0.5^a^	1.0^a^	0.5^a^
11	36	-1.115(2.945)	74	-0.342(2.715)	515	0.293(2.879)	74	-0.830(3.926)	36	-2.116(2.684)	0.3	0.5^a^	0.1	0.4	0.2	0.1	0.7^a^	0.3^a^	0.9^a^	0.4^a^
12	35	-0.743(3.152)	72	-0.111(2.924)	501	0.973(3.455)	72	-0.093(3.334)	35	-1.815(3.213)	0.2	0.5^a^	0.2	0.3	0.3^a^	0.0	0.6^a^	0.3^a^	0.8^a^	0.5^a^
13	34	-2.100(2.630)	70	-1.344(2.720)	485	-0.206(3.049)	70	-0.681(3.661)	34	-1.756(3.883)	0.3	0.7^a^	0.5^a^	0.1	0.4^a^	0.2	0.1	0.1	0.4^a^	0.3
14	36	-1.838(2.590)	74	-1.240(2.309)	516	0.406(3.172)	74	-0.075(3.916)	36	-0.627(3.715)	0.2	0.8^a^	0.5^a^	0.4	0.6^a^	0.4^a^	0.2	0.1	0.3	0.1
15	37	-1.493(2.954)	74	-1.015(2.823)	524	1.001(2.992)	69	-0.364(3.679)	37	-0.528(3.194)	0.2	0.8^a^	0.3	0.3	0.7^a^	0.2	0.2	0.4^a^	0.5^a^	0.1
16	37	-2.629(2.159)	75	-0.846(3.059)	526	0.305(2.779)	75	-0.595(2.614)	37	-2.618(3.153)	0.7^a^	1.2^a^	0.9^a^	0.0	0.4^a^	0.1	0.6^a^	0.3^a^	1.0^a^	0.7^a^
17	36	-2.166(2.731)	74	-1.136(2.773)	513	0.499(2.783)	74	-0.708(3.086)	36	-1.562(3.113)	0.4	1.0^a^	0.5^a^	0.2	0.6^a^	0.2	0.1	0.4^a^	0.7^a^	0.3
18	36	-2.152(2.798)	74	-0.121(2.573)	516	0.719(2.699)	74	-0.707(2.899)	36	-2.096(2.888)	0.8^a^	1.0^a^	0.5^a^	0.0	0.3^a^	0.2	0.7^a^	0.5^a^	1.0^a^	0.5^a^

Note

^#^ effect size between different groups

^a^ *P <* 0.05.

**Table 4:** Comparisons of physical fitness index among girls in different body mass index and age groups.

Age(yr)	BMI<5th Percentile(A)		5th≤BMI<15th Percentile(B)		15th≤BMI<85th Percentile(C)		85th≤BMI<95th Percentile(D)		BMI≥95th Percentile(E)		Cohen’s *d* ^*#*^									
	N	Mean(SD)	N	Mean(SD)	N	Mean(SD)	N	Mean(SD)	N	Mean(SD)	A/B	A/C	A/D	A/E	B/C	B/D	B/E	C/D	C/E	D/E
7	37	-2.147(2.559)	76	-1.543(2.666)	528	-0.174(2.946)	76	-0.039(2.762)	37	-0.886(3.427)	0.2	0.7^a^	0.8^a^	0.4	0.5^a^	0.6^a^	0.2	0.0	0.2	0.3
8	36	-0.580(3.612)	74	-0.514(3.239)	514	-0.030(2.998)	74	0.505(2.851)	36	0.608(3.201)	0.0	0.2	0.3	0.3	0.2	0.3^a^	0.3	0.2	0.2	0.0
9	36	-0.210(3.180)	74	0.316(3.046)	517	0.450(3.312)	74	0.666(2.788)	36	0.329(3.523)	0.2	0.2	0.3	0.2	0.0	0.1	0.0	0.1	0.0	0.1
10	37	-1.544(3.642)	76	-1.313(2.702)	531	-0.210(3.054)	76	-0.803(3.077)	37	-0.959(3.312)	0.1	0.4^a^	0.2	0.2	0.4^a^	0.2	0.1	0.2	0.2	0.0
11	32	-1.134(3.383)	66	-0.429(2.483)	460	-0.021(3.017)	66	0.844(3.195)	32	0.019(4.528)	0.2	0.3^a^	0.6^a^	0.3	0.1	0.4^a^	0.1	0.3^a^	0.0	0.2
12	36	-0.906(2.555)	72	-0.091(2.651)	509	0.686(2.820)	72	0.951(2.976)	36	0.137(3.065)	0.3	0.6^a^	0.7^a^	0.4	0.3^a^	0.4^a^	0.1	0.1	0.2	0.3
13	36	-1.119(3.008)	74	-1.14(3.325)	518	0.074(3.120)	74	-0.729(3.316)	36	-1.140(2.869)	0.0	0.4^a^	0.1	0.0	0.4^a^	0.1	0.0	0.2^a^	0.4^a^	0.1
14	36	-0.502(2.726)	73	-0.292(3.277)	511	0.126(2.930)	73	0.284(2.871)	36	-1.419(3.564)	0.1	0.2	0.3	0.3	0.1	0.2	0.3	0.1	0.5^a^	0.5^a^
15	36	-0.148(2.662)	74	0.360(2.517)	513	0.335(3.059)	74	0.323(3.086)	36	-0.848(3.263)	0.2	0.2	0.2	0.2	0.0	0.0	0.4^a^	0.0	0.4^a^	0.4
16	37	-1.277(2.820)	76	-0.886(3.419)	528	-0.153(3.011)	76	-0.355(3.236)	37	0.014(3.434)	0.1	0.4^a^	0.3	0.4	0.2	0.2	0.3	0.1	0.1	0.1
17	52	0.181(3.126)	61	0.483(3.042)	532	0.118(2.686)	76	-0.164(2.672)	37	-0.075(2.877)	0.1	0.0	0.1	0.1	0.1	0.2	0.2	0.1	0.1	0.0
18	37	0.092(2.716)	74	0.438(2.683)	521	0.183(2.784)	74	0.496(2.425)	37	-0.872(2.285)	0.1	0.0	0.2	0.4	0.1	0.0	0.5^a^	0.1	0.4^a^	0.6^a^

Note

^#^ effect size between different groups

^a^ *P <* 0.05.

In boys aged 8 and 10–18 years, the PFI of those with normal BMI was significantly higher than those with very low BMI (*P* < 0.05). In boys aged 7, 9–13, and 15–18 years, the PFI of the normal BMI group was significantly higher than that of the very high BMI group (*P* < 0.05). In boys aged 8, and 12–18 years, the PFI of the normal BMI group was significantly higher than that of the low BMI group (*P* < 0.05). In boys aged 9–12 and 15–18 years, the PFI of the normal BMI group was significantly higher than that of the high BMI group (*P* < 0.05). In boys aged 7, 9, and 10 years, the PFI of the very low BMI group was significantly higher than that of the very high BMI group (*P* < 0.05). In boys aged 7–18 years, the average PFI of those with normal BMI was higher than the other four BMI groups. These differences varied by age, as did the effect size (Table 3). As BMI increased in boys aged 7–18 years, PFI first increased and then decreased; it reached the peak when BMI was in the normal range (Fig 1). Overall, a BMI below or above the normal range in boys aged 7–9 years had a relatively small impact on PFI. In contrast, a BMI below or above the normal range in boys aged 10–18 years had a significant impact on PFI.

For girls aged 7–9 years, the PFI of those with high BMI was higher than the other BMI groups. In girls aged 10–14 years, the PFI in the normal BMI group was higher than the other BMI groups. The highest PFI values were observed for girls aged 15–18 years with low BMI and girls aged 16 years with very high BMI. In girls aged 7, 10, and 16 years, the PFI of the normal BMI group was significantly higher than the very low BMI group. In girls aged 13–15 and 18 years, the PFI of the normal BMI group was significantly higher than the very high BMI group, with different effect sizes (Table 4). Overall, as BMI increased, the PFI of girls aged 7–15 years showed a trend of first increasing and then decreasing, but the fluctuation was smaller than that observed for boys. The effect of BMI on PFI in girls aged 16–18 years varied significantly (Fig 1).

**Fig 2:**
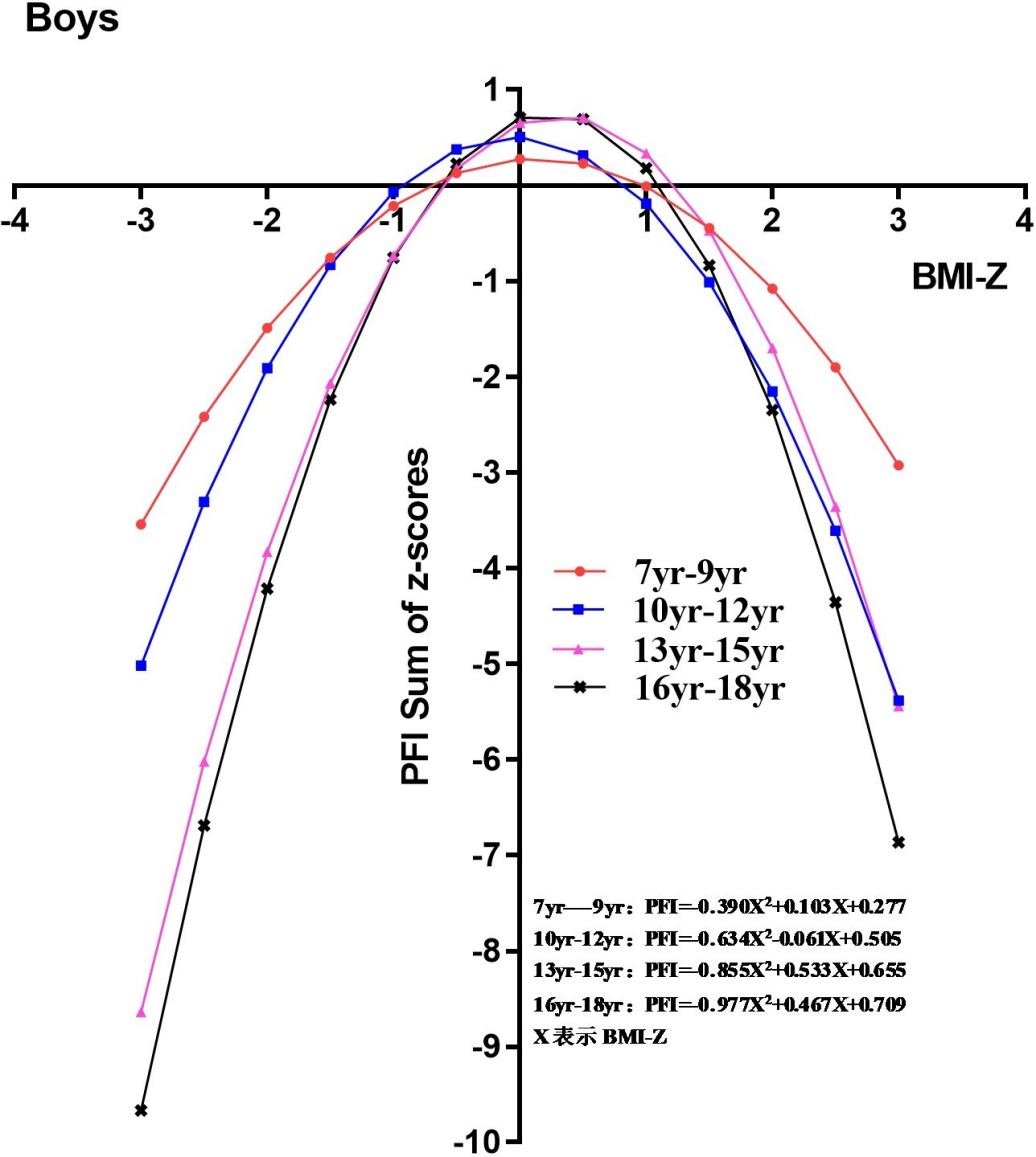
Linear regression analysis of physical fitness index and body mass index for boys in different age groups.

**Fig 3:**
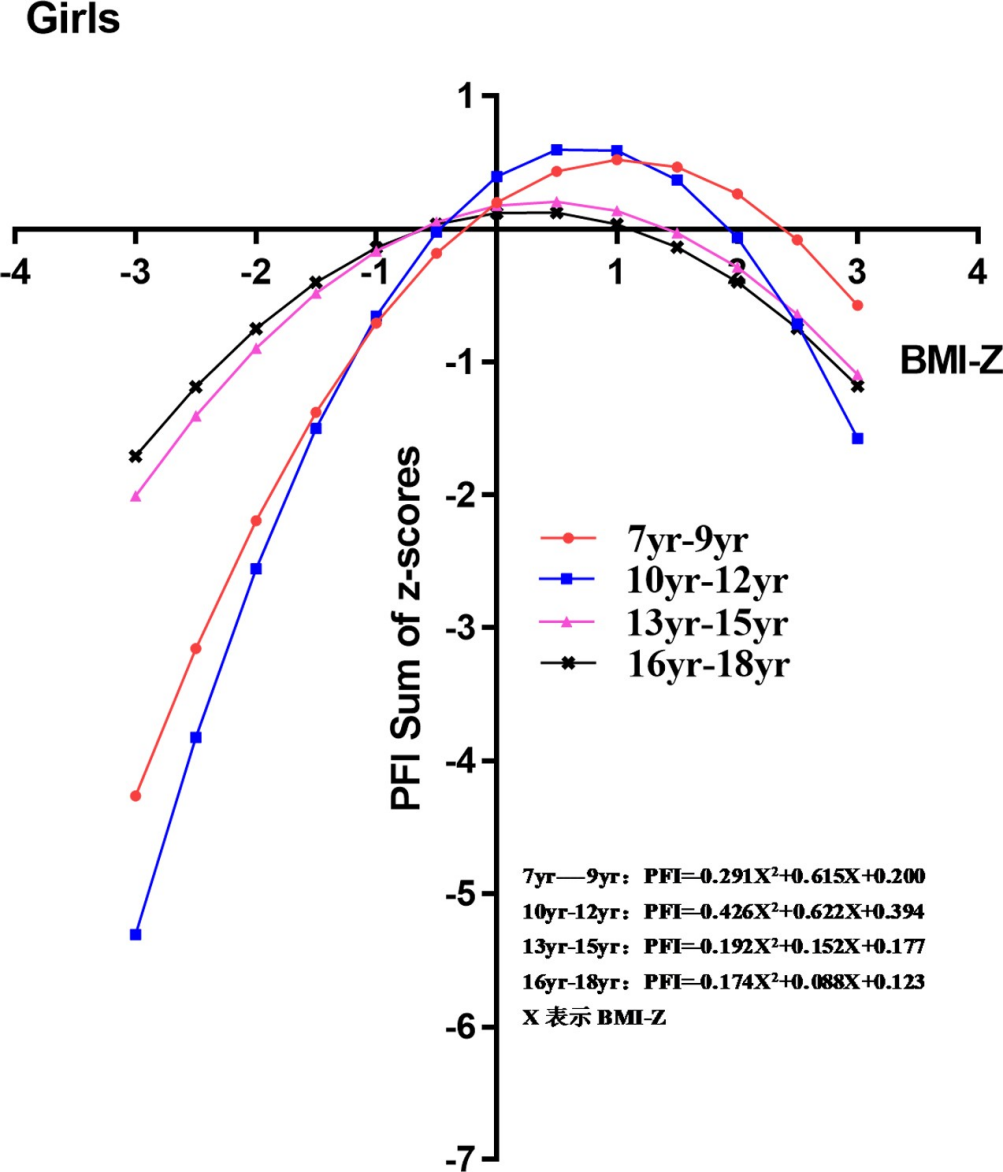
Linear regression analysis of physical fitness index and body mass index for girls in different age groups.

The nonlinear regression analysis showed regression equations and parabolic trend graphs of the relationship between BMI Z-score and PFI for boys and girls in all age groups (Figs 2 and 3). Compared with boys aged 13–15 and 16–18 years, variations in PFI with the increase in BMI Z-scores for boys aged 7–9 and 10–12 years were smaller. PFI reached its peak when BMI was in the normal range for boys in all age groups (Fig 2). Among girls, there was large variation in PFI when the BMI Z-score was in the normal range. However, when the BMI Z-score was higher than the normal range, the variation in PFI became smaller for girls aged 7–9 and 10–12 years. The result above indicated that changes in BMI among prepubescent girls had a greater impact on PFI. In contrast, the variation in PFI for girls aged 13–15 and 16–18 years was small, irrespective of whether the BMI Z-score was normal or higher than normal (Fig 3).

Figs 2 and 3 suggest that PFI was affected by puberty. The average age of puberty in girls is approximately 1–2 years earlier than in boys. In the present study, very low or low BMI in boys during the pre-puberty period (7–9 and 10–12 years) had no correlation with PFI. In contrast, very low or low BMI in girls during pre-puberty and puberty (7–9, 10–12, and 13–15 years) was significantly associated with PFI. When girls reached age 16–18 years, the association between BMI and PFI gradually diminished. PFI showed a parabolic curvilinear relationship with BMI.

## Discussion

The results of this study showed that PFI had a parabolic trend of first increasing then decreasing as BMI increased in children and adolescents from Xinjiang Uyghur Autonomous Region, China. Children and adolescents with a normal BMI demonstrated good physical fitness, whereas a BMI below or above the normal range appeared to be related to poor physical fitness. The relationship between BMI and PFI showed an inverted U-shaped curve. Ribeiro et al. reported that white girls aged 8–15 years showed a downward trend in VO_2max_ as BMI increased, and the correlation was significant[26]. A study by Malina et al. demonstrated that obesity had a negative effect on physical exercise capacity for American children and adolescents aged 6–14 years, including for long jump, high jump, and sprint[27]. Another study reported that the cardiopulmonary endurance functions of Chinese children and adolescents with normal weight were better than those who were underweight, overweight, and obese, and the relationship between 20 m round-trip Z-scores and BMI showed a parabolic curve trend[28].

In the low and very low BMI groups, boys aged 7–9 and 10–12 years showed a small increase in PFI as BMI increased, whereas girls in these age groups showed a large increase. In the high and very high BMI groups, changes in BMI had a significant impact on PFI for both boys and girls. We also showed that the effects of BMI on PFI in the pre-puberty period were more pronounced in girls than in boys. A previous study demonstrated that puberty timing and sexual development was significantly earlier in girls than boys, resulting in a greater correlation between BMI and physical fitness in girls than boys [29, 30].

Sekulic et al.[31] concluded that for college boys, the relationship between BMI and push-ups, sit-ups, high jump, 50 m swim, and 1,500 m run showed a nonlinear trend, which was consistent with the results obtained in this study. Lu et al. also showed that the relationship between PFI and BMI of adult men conforms to the quadratic model, and the influence of PFI by BMI is more serious in middle-aged male than youth male [18].

The results of the present study showed that the best performance was observed when BMI was 20.2–22.2 kg/m^2^, and the worst performance was observed when BMI was at the lowest (19.0–19.7 kg/m^2^) and highest (24.0–27.0 kg/m^2^) levels. The maximum PFI score (1.001, corresponding to the best physical fitness) was observed in boys aged 15 years with a BMI in the normal range (17.47–22.35 kg/m^2^). In girls, the maximum PFI (0.686) was observed in those aged 12 years with BMI in the normal range (15.72–21.61 kg/m^2^). These results support earlier puberty development in girls than boys, and suggest the optimal age for good physical fitness may be younger in girls than in boys.

Overall, as age increased, we observed BMI had a significant impact on PFI in adolescent boys (13–18 years) and pre-pubescent and adolescent girls (7–15 years). After puberty (16–18 years), BMI showed a weak association with PFI, and was weaker in girls than in boys (Figs 2 and 3). Previous studies have shown that BMI increased during puberty, with boys mostly gaining muscle mass and girls mostly gaining fatty tissue[32–34]. Some studies involving the step cardiopulmonary endurance test showed no significant differences among female college students with different obesity levels, whereas male college students showed a significant decline in test scores with increased obesity levels[35–36].

Overall, there was a parabolic relationship between BMI and PFI in children and adolescents from Xinjiang, China. Before puberty, the influence of BMI on PFI was more significant in girls than boys. However, after puberty, the association between BMI and PFI was more significant in boys than in girls.

Currently, the Xinjiang region is working toward a health goal of improving the physical fitness of children and adolescents in multi-ethnic regions. The present results suggest there is need to accelerate public health efforts in developing and implementing school-based physical education policies to improve students’ physical fitness and health. For example, the health implications of lower fitness could be emphasized in the physical education curriculum to raise students’ awareness. In addition, the customized implementation of nutrition improvement programs may be helpful for children and adolescents with low BMI levels, whereas suitable obesity intervention programs are warranted to support effective weight loss for those with high BMI levels.

Our study had several limitations. First, the data were drawn from the 2014 CNSSCH, which was conducted over five years ago; however, these data are the most recent and authoritative data available. Second, this was a cross-sectional study and therefore cannot make conclusions as to whether obesity caused low fitness or vice versa. Third, we classified weight status using BMI. However, BMI is an index of relative weight rather than body fat, and cannot differentiate the levels of fatness and leanness among individuals.
